# A Community-Based Study of Quality of Life and Depression among Older Adults

**DOI:** 10.3390/ijerph13070693

**Published:** 2016-07-09

**Authors:** Wenjun Cao, Chongzheng Guo, Weiwei Ping, Zhijun Tan, Ying Guo, Jianzhong Zheng

**Affiliations:** 1 Department of Preventive Medicine, Chang Zhi Medical College, Changzhi 046000, China; gczmed@126.com (C.G.); czmeeting2013@126.com (W.P.); 2 Department of Health Statistics, Fourth Military Medical University, Xi’an 710032, China; tanzjfmmu@126.com; 3 Institute for Cardiovascular Disease, Chang Zhi Medical College, Changzhi 046000, China; 18bbz@163.com

**Keywords:** quality of life, depression, elderly Chinese, WHOQOL-BREF, Geriatric Depression Scale

## Abstract

The goal of the study was to assess the quality of life (QOL) and depression and provide further insights into the relationship between QOL and depression among community-dwelling elderly Chinese people. Baseline data were collected from 1168 older adults (aged ≥ 60) in a large, prospective cohort study on measurement and evaluation of health-promoting and health-protecting behaviors intervention on chronic disease in different community-dwelling age groups. QOL was assessed using the 26-item, World Health Organization Quality of Life, brief version (WHOQOL-BREF) and depression was assessed using the 30-item Geriatric Depression Scale (GDS). The mean WHOQOL-BREF score for all dimensions was approximately 60, with the highest mean value (61.92) observed for social relationships, followed by environment, physical health, and psychological health domains. In this cohort, 26.1% of elderly urban adults met GDS criteria for depression. There were negative correlations between physical health (Odds Ratio (OR) = 0.928, 95% Confidence Interval (CI): 0.910–0.946), psychological health (OR = 0.906, 95% CI: 0.879–0.934), environment (OR = 0.966, 95% CI: 0.944–0.989) and depression among elderly people. Those with depression were older, less educated, had a lower monthly income, and were more likely to report insomnia. All WHOQOL-BREF domains, with the exception of the social domain were negatively correlated with depression.

## 1. Introduction

With the increased life expectancy for older people, there has also been an increased interest in the assessment of quality of life (QOL), and depression in later stages of life [1]. The World Health Organization (WHO) has defined QOL as a person’s perception of his or her life position in the value system and the culture in which they live, and in addition, it is related to one’s life goals, expectations, standards and concerns [2]. The WHO Quality of Life (WHOQOL) group has developed a brief QOL assessment scale, the World Health Organization Quality of Life, brief version (WHOQOL-BREF), which consists of 26 items representing four domains: physical, psychological, social and environmental [3]. Psychometric studies have indicated that the WHOQOL-BREF has cross-cultural validity as a QOL assessment tool [3]. In China, the psychometric properties of the WHOQOL-BREF have been tested, and good reliability and validity demonstrated for Chinese people [4]. 

QOL of older adults has become an important issue, because of demographic changes resulting from the ageing of the population. Moreover, studies have suggested that QOL scores of elderly people are different from that of the general population [5]. Furthermore, although the QOL has been a focus of attention for over a decade, there are few recent data available on the QOL of the elderly.

Depression is another important public health problem for older adults, because late life depression might have devastating consequences, such as an increase in mortality [6]. The WHO estimates that worldwide prevalence of geriatric depression varies between 10% and 20% [7]. In China, especially in recent years, this rate has been significantly high. In our previous studies, 27% of urban elderly adults (aged ≥ 60) [8] and 30.8% of rural elderly adults (aged ≥ 65) have reported symptoms of depression [9]. The Geriatric Depression Scale (GDS) is a 30-item, self-report instrument used for measuring depression among the elderly, which has excellent validity. The GDS is a useful instrument for screening elderly Chinese populations [10]. There are no firm conclusions regarding whether depression among the elderly is associated with demographic variables. Therefore, one aim of this study was to assess the prevalence of elderly in China and examine possible associations between the elderly and demographic variables.

Mental health has been identified as one of the most important factors in QOL [11]. Mental illnesses are known to be increasing, and account for 15% of the total disease burden in developing countries [12]. Depression is considered to be the most common mental health problem among older people. The degree of suffering caused by depression is not easy to assess, although one possible and effective method of assessing the suffering caused by depression is to evaluate its impact on QOL [13]. Recently, limited knowledge has accumulated on the association between the QOL and depression among elderly Chinese people dwelling in the community. 

It is estimated that about 15.2% of the total population of China was over 60 years of age in 2015, indicating that the whole population is aging [14]. Therefore, investigating QOL and depression among older adults is important. In the present study, we examined the quality of life, the prevalence of depression and possible influence of demographic variables, and the association between QOL and depression among elderly Chinese people, by sampling a large, community-based, elderly population (aged ≥ 60). 

## 2. Methods

### 2.1. Study Design and Sample

The survey was conducted as a part of a large, prospective, cohort study on measurement and evaluation of health-promoting and health-protecting behaviors intervention on chronic disease in different community-dwelling age groups [15]. We used the baseline data of the larger study and examined the quality of life, the prevalence and risk factors of depression, and the association between the QOL and depression. 

A survey was conducted from May 2014 to September 2015 with elderly individuals aged 60 years or older living in Changzhi. Changzhi is a historic city, which is also known as the “Shangdang Basin”. It is located in the southeast of Shanxi Province, covers a geographic area of 334 km^2^, and has a population of 556,100. The average income of the people in this area is slightly below the national average. There are three districts in the city of Changzhi 2015, which include urban, suburban and high-tech areas. 

Multistage stratified cluster sampling was used in this study. We selected an urban area; then, three streets were randomly selected from this area, and finally one or two communities were randomly selected from each street. All the people aged 60 years and above in the five selected communities were invited to participate in the survey. We excluded individuals with cognitive impairments, those requiring long-term care, those dwelling less than 5 years in the current community, and those that were not available during the study period. A total of 1265 urban elderly residents were recruited, among which 1188 completed the questionnaires. Of these, 1168 questionnaires that had no missing values in WHOQOL-BREF and GDS were included in the analysis. The data were collected through face to face interviews by trained investigators. 

### 2.2. Assessment Instruments

#### 2.2.1. WHOQOL-BREF

The WHOQOL-BREF was used to assess the QOL of elderly adults. The WHOQOL-BREF comprises of 26 items, 24 of these items are divided into four domains (Physical, Psychological, Social and Environmental), with two individual items assessing the perception of overall QOL and general health [4]. All items are rated on a 5-point scale, with higher scores indicative of higher QOL. The raw score for each of the four WHOQOL-BREF dimensions are derived by summing the item scores and transforming them to a scale ranging from 0 to 100 using the formula below [16]:

(1) $$\text{Transformed scale}=\left[\frac{\left(\text{Actual raw score}-\text{Lowest possible raw score}\right)}{\text{Possible raw score range}}\right]\;\times \;100$$

Lower scores indicate poor QOL. Scores over one standard deviations (SD) below the mean were considered as indicative of poor QOL [4]. This questionnaire had sufficient internal consistency in this study, as indicated by a Cronbach’s α score of 0.864.

#### 2.2.2. GDS

The 30-item Chinese version of the GDS was used to identify depressive symptoms in older individuals. This scale has been specially developed for assessing depression in older adults. The scale has been widely used in different settings, including in community studies [8]. Participants responded with “Yes” or “No” to each item of the scale. A scores of one was assigned to each positive response, such that the maximum possible score for the GDS was 30. A score of 11 or above in the GDS is considered to indicate depression [17]. The GDS is easy to use and has high sensitivity (70.6%) and specificity (70.1%) for assessing older Chinese people aged 60 years or above [18]. The GDS has also demonstrated sufficient internal consistency in this study, as indicated by a Cronbach’s α score of 0.642.

#### 2.2.3. Other Variables

In addition to the above variables, key demographic, social, and individual characteristics, including age, gender, education, monthly income, current marital status and body mass index (BMI, derived from height and weight measurements) were collected. 

### 2.3. Statistical Analyses

All questionnaires were numbered. A double data entry procedure was conducted by two trained data-entry workers using Epidata software 3.1 (EpiData Association, Odense, Denmark). Both computer and manual controls were used to ensure data accuracy. All data were analyzed using SPSS version 18.0 (SPSS, Inc., Chicago, IL, USA). Descriptive statistics such as frequencies, percentages, means, and SD were calculated to assess sample characteristics. A Chi-square test was conducted to determine significant differences between depression and demographic variables. A logistic regression analysis, which allows simultaneous testing of associations between two or more explanatory variables, was conducted to explore relationships between depression and WHOQOL-BREF domains, after adjusting for age, education level, monthly income, and self-reported insomnia. A two-tailed *p*-value less than 0.05 (*p* < 0.05) was considered statistically significant in all analyses.

### 2.4. Ethical Aspects

All subjects gave their informed consent for inclusion before they participated in the study. The study was conducted in accordance with the Declaration of Helsinki, and the protocol was approved by the Ethics Committee of Chang Zhi Medical College (2016052). 

## 3. Results

### 3.1. Sample Characteristics

The response rate in this study was 92.3% (1168 of 1265 respondents). The characteristics of the sample are shown in Table 1. It can be seen that the mean age of participants was 70.70 years (SD = 7.07, range 60–94). Among the 1168 participants, 556 (47.6%) were men and 612 (52.4%) were women. Health-related factors of participants are also presented in Table 1. As can be seen from the table, approximately 30% of the elderly people had self-reported insomnia, about 70% reported chronic diseases, and more than half were overweight based on cut-off score of the BMI for Chinese samples (BMI ≥ 24).

### 3.2. Depression in the Elderly Population

In this study, “depression” was operationally defined as having a GDS score of 11 or above. As shown in Table 2, the respondents comprised 863 participants (73.9%) without depression and 305 (26.1%) with depression. Table 2 shows the comparison between depression and non-depression groups, which indicated no significant differences in gender, current work status, marital status, BMI, or self-reported chronic diseases between the two groups. However, age (*χ*^2^ = 8.7, *p* < 0.05), education level (*χ*^2^ = 11.9, *p* < 0.05), self-reported insomnia (*χ*^2^ = 11.6, *p* < 0.05), and monthly income (*χ*^2^ = 8.6, *p* < 0.05) were significantly associated with depression. People with depression were older, less educated, had a lower monthly income, and were more likely to report insomnia. 

### 3.3. Quality of Life in the Elderly Population

Table 3 shows means, SDs and raw scores for each item of WHOQOL-BREF. Three items (Q3, Q4 and Q26) were reverse scoring items. Table 4 shows WHOQOL-BREF domain scores. Each domain score was transformed to a scale ranging from 0 to 100, to enable comparisons between different domains consisting of unequal numbers of items. The average scores of all WHOQOL-BREF dimensions were approximately 60. The highest mean value (61.92) was observed for the social relationships domain, followed by environmental, physical health, and psychological health domains. We defined a score of one SD below the mean as the cut-off point for poor QOL, based on a previous study [4]. Table 4 shows the proportion of elderly people below the cut-off score. It can be seen that 11.6 (Psychological Health)-28.8% (Social Relationships) of the sample in this study was had poor QOL.

### 3.4. Correlation between the QOL and Depression

A logistic regression analysis was performed to explore the relationship between depression and WHOQOL-BREF domains. Table 5 presents both the crude and the adjusted odds ratios for depressive symptoms in WHOQOL-BREF domains. The odds ratios were adjusted for age, education level, monthly income, and self-reported insomnia, which indicated significant differences in the univariate analysis (*p* < 0.05). The results showed negative correlations between physical health (Odds Ratio (OR) = 0.928, 95% Confidence Interval (CI): 0.910–0.946), psychological health (OR = 0.906, 95% CI: 0.879–0.934), environment (OR = 0.966, 95% CI: 0.944–0.989) and depression among elderly people, which were significant after controlling for age, education level, monthly income, and self-reported insomnia. 

## 4. Discussion

The present study assessed the QOL of older people and investigated differences in QOL between elderly people with and without depressive symptoms in a moderately large sample. QOL of elderly Chinese people was evaluated by the WHOQOL-BREF, which has been demonstrated to be a suitable instrument for assessing community-dwelling older populations [19]. The mean score for overall QOL in this study was 62.15, which was higher than in people with hypertension [20] and type 2 diabetes [21]. However, a different study has reported a slightly higher mean score compared to the current study for overall QOL in 583 health-care staff [22]. The highest and the lowest mean scores of the four domains of WHOQOL-BREF in the current study were 61.92 for social relationships domain and 58.41 for the psychological health domain, which was similar to findings of a study on a population of older adult [20]. Moreover, we found that 11.6%–28.8% of urban elderly had low QOL, compared to 6.9%–15.9% of urban adults aged 18/18+ years [4]. Regardless of the criterion that was used, this study found that elderly Chinese people had relative low QOL compared to data reported in Western countries [22,23]. This was particularly the case in psychological, social, and environmental domains [24]. The results also indicated that the domain scores in our study were higher than those in India [25,26]. This suggests the possibility that QOL scores might be related to average income of the citizens of a country.

Depression is known to be the most common psychological disorder in older adults. The average prevalence of depression in elderly individuals in the world has been estimated as 10.3% [27]. In the United States, approximately 15% to 20% of older adults suffer from depression each year [28]. Numerous studies have indicated that the depression rate among older adults is significantly higher in Asia [29,30]. Studies that have investigated the prevalence of depression among older people in Mainland China have reported depression rates ranging from 15.8% to 30.8%, probably due to the use of different methodologies [9,31,32,33]. In our previous study, we reported that the prevalence of depression was 27.0% among older urban people in Western China [8], compared to 26.1% in northern Chinese population sampled in the current study using identical assessment methods. 

The univariate analysis in this study indicated significant associations between age, income level, education, insomnia, and depression. An age-related increase in the prevalence of depression was also found in Stordal’s research, which was consistent with the current study [34]. Previous studies have indicated a significant effect of education and income levels on the risk for depression [35,36], which was confirmed in this study. Our findings also support the association between depression and insomnia similar to a Norwegian study, which reported a bidirectional association [37]. 

Consistent with previous studies [38], all WHOQOL-BREF domains, with the exception of the social domain were negatively correlated with depression. These negative correlations remained after adjusting for age, education level, monthly income, and self-reported insomnia, which showed significant differences in the univariate analysis. The mean scores of the participants with depression in all four WHOQOL-BREF domains were lower than those of participants without depression. Although no significant association was detected between social domain and depression, elderly people having depression in the social domain scored approximately 10 points (55.03 vs. 64.36) lower than non-depressed participants. One previous study that examined associations between depression and QOL among Chinese elderly in Hong Kong [13], reported that scores on all four WHOQOL-BREF domains were significantly associated with depression.

This study is a much larger study than those that have been conducted previously. Moreover, the response rate of 92% in this survey is very high. It is generally known that results obtained from bigger samples having a higher response rate are more robust. Nevertheless, certain limitations of this study might constrain the validity of its findings. Most importantly, we adopted the Chinese version of the 30-item GDS, rather than using diagnostic criteria for clinical depression in defining depression. As a result, the prevalence of depression in this study could be overestimated. Additionally, the cross sectional study design cannot suggest causal relationships. Therefore, it is suggested that a longitudinal study should be conducted in the future, in order to estimate the direction of causation between WHOQOL-BREF domains and depression. 

## 5. Conclusions

This is one of the largest studies to investigate associations between QOL and depression. This study provides further insights into associations between QOL and depression among community-based, elderly Chinese people. All the WHOQOL-BREF domains, with the exception of the social domain were negatively correlated with depression. The prevalence of depression in our sample was 26.1%. Moreover, people with depression were older, less educated, had a lower monthly income, and were more likely to report insomnia. 

## Figures and Tables

**Table 1:** Demographic characteristics of the sample (*n* = 1168).

Variable	*N*	Percentage
Age (years)		
60–69	583	49.9
70–79	427	36.6
80 or above	158	13.5
Gender-female	612	52.4
Education		
No formal education	91	7.8
Primary school	236	20.2
Junior high school	378	32.4
Senior high school/technical secondary school	262	22.4
Post-secondary and above	201	17.2
Monthly income (in RMB)		
Less 1000 ($154.7)	208	17.9
1000–2999	530	45.7
3000 ($464.1) and above	421	36.3
Currently married	943	80.7
Currently working	78	6.7
Self-reported insomnia	372	31.8
Self-reported chronic diseases	780	66.8
Body Mass index (Kg/m^2^)		
<18.5	51	4.4
18.5–23.9	522	44.7
24.0–27.9	481	41.2
≥28.0	114	9.8

**Table 2:** Demographic variables related to depression among older people (*n* = 1168).

Variable	Depression		Non-Depression		*χ*^2^	*p*-Value
	*N*	%	*N*	%		
Total	305	26.1	863	73.9		
Age (years)						
60–69	131	43.0	452	52.4	8.728	0.013
70–79	123	40.3	304	35.2		
80 or above	51	16.7	107	12.4		
Gender-female	163	53.4	449	52.0	0.181	0.671
Education						
No formal education	36	11.8	55	6.4	11.932	0.008
Primary school	68	22.3	168	19.5		
Junior high school	91	29.8	287	33.3		
Senior high school/technical secondary school	59	19.3	203	23.5		
Post-secondary and above	51	16.7	150	17.4		
Monthly income (in RMB)						
Less 1000 ($154.7)	73	24.3	135	15.7	12.087	0.002
1000–2999	120	39.9	410	47.8		
3000 ($464.1) and above	108	35.9	313	36.5		
Missing	4		5			
Currently married	235	77.0	708	82.0	3.608	0.057
Currently working	18	5.9	60	7.0	0.399	0.527
Self-reported insomnia	121	39.7	251	29.1	11.638	0.001
Self-reported chronic diseases	201	65.9	579	67.1	0.144	0.705
Body Mass index (Kg/m^2^)						
<18.5	14	4.6	37	4.3	1.820	0.611
18.5–23.9	140	45.9	382	44.3		
24.0–27.9	115	37.7	366	42.4		
≥28.0	36	11.8	78	9.0		

**Table 3:** Scores of WHOQOL-BREF items among the elderly Chinese (*n* = 1168).

WHOQOL-BREF Items/Domains	Direction of Scaling	Mean Raw Item Score	Standard Deviation (SD)
Q1 Overall QOL	+	3.55	0.69
Q2 General health	+	3.42	0.76
**Domain 1: Physical Health**			
Q3 Physical pain	−	2.48	0.79
Q4 Dependence medication	−	2.63	0.85
Q10 Energy	+	3.16	0.71
Q15 Mobility	+	3.18	0.80
Q16 Sleep and rest	+	3.31	0.89
Q17 Activities of daily living	+	3.51	0.68
Q18 Working capacity	+	3.45	0.70
**Domain 2: Psychological Health**			
Q5 Life enjoyment	+	3.12	0.55
Q6 Meaningfulness of life	+	3.21	0.59
Q7 Concentration	+	3.11	0.64
Q11 Body appearance	+	3.25	0.58
Q19 Self-esteem	+	3.65	0.63
Q26 Negative feelings	−	2.33	0.84
**Domain 3: Social Relationships**			
Q20 Personal relationship	+	3.60	0.63
Q21 Sexual activity	+	3.25	0.84
Q22 Social support	+	3.58	0.60
**Domain 4: Environment**			
Q8 Safety	+	3.30	0.59
Q9 Physical environment	+	3.31	0.61
Q12 Financial resources	+	3.21	0.81
Q13 Daily information	+	3.20	0.66
Q14 Leisure	+	3.07	0.82
Q23 Home environment	+	3.72	0.67
Q24 Access to health care	+	3.65	0.64
Q25 Transport	+	3.55	0.69

**Table 4:** Scores of the WHOQOL-BREF subscales among Chinese elderly (*n* = 1168).

WHOQOL-BREF Domains	Item Amount	Minimum	Maximum	Mean	SD	Poor QOL	
						*N*	%
Physical Health	7	10.7	100.0	59.00	12.4	198	17.0
Psychological Health	6	4.2	100.0	58.41	10.9	136	11.6
Social Relationships	3	16.7	100.0	61.92	11.5	336	28.8
Environment	8	21.9	100.0	59.37	11.2	141	12.1

**Table 5:** Logistic regression analysis of QOL and depression.

WHOQOL-BREF Domains (Every 1-Point Increase)	Depression (Mean ± SD)	Non-Depression (Mean ± SD)	Crude Odds Ratio (95% Confidence Interval)	*p*-Value	Adjusted Odds Ratio (95% Confidence Interval)	*p*-Value *
Physical Health	48.80 ± 11.71	62.59 ± 10.44	0.928 (0.910–0.946)	<0.001	0.928 (0.908–0.948)	<0.001
Psychological Health	49.84 ± 10.54	61.44 ± 9.31	0.906 (0.879–0.934)	<0.001	0.903 (0.873–0.933)	<0.001
Social Relationships	55.03 ± 11.20	64.36 ± 10.63	1.016 (0.994–1.038)	0.147	1.007 (0.984–1.031)	0.567
Environment	51.85 ± 11.28	62.03 ± 9.82	0.966 (0.944–0.989)	0.004	0.972 (0.948–0.997)	0.030

* Adjusted for age, education, monthly income, and self-reported insomnia, which were noted as significant in the univariate analysis.
